# Short-term effect of particulate matter on lung function and impulse oscillometry system (IOS) parameters of chronic obstructive pulmonary disease (COPD) in Beijing, China

**DOI:** 10.1186/s12889-023-16308-0

**Published:** 2023-07-24

**Authors:** Rui-xia Zhu, Xiu-hong Nie, Xiao-fang Liu, Yong-xiang Zhang, Jin Chen, Xue-jiao Liu, Xin-jie Hui

**Affiliations:** 1 Department of pulmonary and critical care medicine, Xuanwu Hospital, Capital Medical University, Beijing, China; 2 Department of pulmonary and critical care medicine, Tong Ren Hospital, Capital Medical University, Beijing, China; 3 Department of pulmonary and critical care medicine, Daxing District People’s Hospital, Beijing, China; 4 Respiratory department, Fuxing Hospital, Capital Medical University, Beijing, China

**Keywords:** Particulate matter, Chronic obstructive pulmonary disease (COPD), Lung function, Impulse Oscillometry System (IOS)

## Abstract

**Objective:**

This study aimed to evaluate the associations between particulate matter (PM), lung function and Impulse Oscillometry System (IOS) parameters in chronic obstructive pulmonary disease (COPD) patients and identity effects between different regions in Beijing, China.

**Methods:**

In this retrospective study, we recruited 1348 outpatients who visited hospitals between January 2016 and December 2019. Ambient air pollutant data were obtained from the central monitoring stations nearest the participants’ residential addresses. We analyzed the effect of particulate matter with aerodynamic diameter ≤ 2.5 μm (PM_2.5_) exposure on lung function and IOS parameters using a multiple linear regression model, adjusting for sex, smoking history, education level, age, body mass index (BMI), mean temperature, and relative humidity .

**Results:**

The results showed a relationship between PM_2.5_, lung function and IOS parameters. An increase of 10 µg/m^3^ in PM_2.5_ was associated with a decline of 2.083% (95% CI: −3.047 to − 1.103) in forced expiratory volume in one second /predict (FEV_1_%pred), a decline of 193 ml/s (95% CI: −258 to − 43) in peak expiratory flow (PEF), a decline of 0.932% (95% CI: −1.518 to − 0.342) in maximal mid-expiratory flow (MMEF); an increase of 0.732 Hz (95% CI: 0.313 to 1.148) in resonant frequency (F_res_), an increase of 36 kpa/(ml/s) (95% CI: 14 to 57) in impedance at 5 Hz (Z_5_) and an increase of 31 kpa/(ml/s) (95% CI: 2 to 54) in respiratory impedance at 5 Hz (R_5_). Compared to patients in the central district, those in the southern district had lower FEV_1_/FVC, FEV_1_%pred, PEF, FEF_75%_, MMEF, X_5_, and higher F_res_, Z_5_ and R_5_ (p < 0.05).

**Conclusion:**

Short-term exposure to PM_2.5_ was associated with reductions in lung function indices and an increase in IOS results in patients with COPD. The heavier the PM_2.5_, the more severe of COPD.

## Introduction

Chronic obstructive pulmonary disease (COPD) is a major health problem in developed and developing countries. It has high incidence and mortality rates worldwide, is caused by airway and alveolar abnormalities and will be a leading cause of death worldwide by 2030 [1, 2]. Evidence suggests that tobacco smoking and occupational exposure to wood smoke predispose people to COPD [3, 4]. In recent years, additional causal associations have been found among both smokers and nonsmokers, including dust, gas, vapor and biological and chemical exposures [5, 6]. Among these risk factors, particulate matter is now recognized as a course of the accuracy of the test results, depending on many health hazards for respiratory diseases, especially COPD. Ambient particulate matter (PM) with an aerodynamic diameter ≤ 2 0.5 μm (PM_2.5_), capable of penetrating and depositing in the deep lung due to its small size, constitutes a large fraction of the PM present in modern urban atmospheres[7, 8]. Numerous epidemiological studies have demonstrated that short-term and long-term exposure to PM_2.5_ can cause adverse respiratory and cardiovascular outcomes [9–11]. Previous studies have indicated that both acute and long-term exposure to outdoor PM_2.5_ is significantly associated with decreased lung function in both adults and children [12, 13]. Because the complex composition and ambient concentrations of PM_2.5_ vary greatly by country and region, the effect of PM_2.5_ on lung function in COPD patients is limited, and the results are inconsistent. In addition, lung function may not be the most sensitive method for identifying COPD at an early stage [14].

Currently, lung function tests are the most common method for diagnosing COPD. However, the accuracy of the test results depends on the close cooperation of the patients. The Impulse Oscillometry System (IOS) is a method for the diagnosis of pulmonary disease that measures the resistance of airways using waves, and detects airway diseases through the diagnosis of changes in airway resistance. It is not dependent on patient cooperation and is more sensitive in the diagnosis of obstructive lesions of the small airways compared to previous methods [15]. In recent years, effective IOS parameters have been found to be significantly more valid than lung function indices, particularly F_res_ and R_5_. It is helpful in improving the accuracy and sensitivity of the results in patients with COPD [16, 17]. Thus, it is urgent to investigate the effect of exposure to PM_2.5_ on lung function and IOS indices in Beijing, China.

In this study, we collected daily outdoor PM_2.5_ from each participant’s residence to assess the effect of short-term PM_2.5_ on lung function indices and IOS outcomes in patients with COPD from 2016 to 2019 in Beijing, China.

## Methods

### Study design

**Fig. 1:**
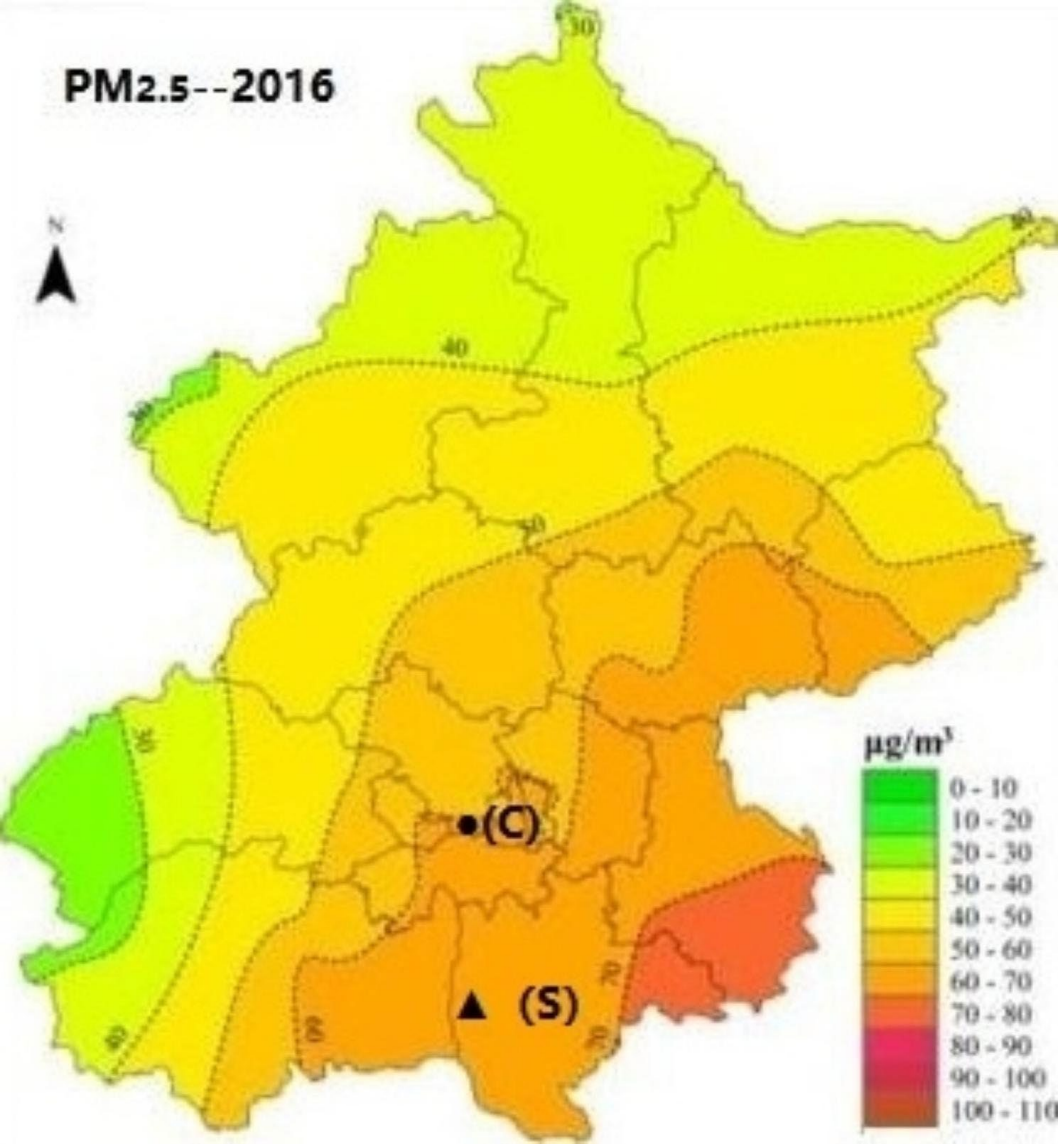
Distribution of all participants regions in 2016 in Beijing. **Abbreviations: PM**_**2.5**,_ particulate matter with aerodynamic diameter ≤ 2.5 μm. ●(C), central district. ▲**(S)**, southern district

According to the Beijing Municipal Environmental Protection Bureau, the pollution level in the southern district of Beijing is higher than that in the central and northern districts (Fig. 1). The southern district has a population of over 1.9 million and is located at 39°26–39°50′N′ and 116°13–116°43′E′. The central district has a population of over 1.1 million and is located at 39°53 − 39°58′N′ and 116°19–116°23′E′. The southern district has a large floating population and serious industrialization. In addition, it is difficult to diffuse and remove PM_2.5_ in the southern district due to its low-lying. The central district belongs to the city center, and there are many tall buildings that can block air flow. Hence, the exposure to PM_2.5_ especially in the southern district is high in the two regions. We recruited participants who were registered as permanent residents from two different districts of Beijing: southern and central. Two general tertiary hospitals in each region were selected for the study. We performed a four-year retrospective observation study based on outpatients who visited hospitals for COPD between January 1, 2016, and December 31, 2019, in the two regions.

The Ethics Committee of Xuanwu Hospital, Capital Medical University, Beijing, China approved the research protocol (No. KS2022170). Written informed consent was waived by the Xuanwu Hospital ethics committee because this was a retrospective multi-center study and it was difficult to obtain consent.

### Participants

Participants were recruited from the outpatient department aged 40–90 years. Information on sex, smoking status, education, time to complete the pulmonary function test, age, height, and weight were included in all COPD patients.

Inclusion criteria (1): The diagnosis of COPD was based on objective measures such as having a post-bronchodilator FEV_1_/FVC < 70% [18]; (2) complete clinical records in the hospital information system (HIS); (3) all the participants lived more than 1000 m away from the main road without exposure to fuels or occupational exposure and (4) no other respiratory complication diseases, such as asthma, lung malignancy, bronchiectasis, pulmonary tuberculosis, or allergic rhinitis.

The exclusion criteria were designed to exclude patients whose lifestyles or complications had a significant impact on their lung function. The exclusion criteria were as follows: (1) the presence of complications, such as severe cardiovascular and cerebrovascular diseases, hepatic and renal insufficiency, and active tuberculosis; and (2) patients with severe COPD who spend few days outside.

### Lung function tests and impulse Oscillometry System

Lung function tests and IOS tests were conducted by trained and certified technicians on participants in a sitting position with a nose clip using a spirometer from the MasterScreen Pneumo PC (JAEGER, Germany and Sensor Medics, USA) in hospitals. IOS tests were performed 10 min after the pulmonary function test. Data were collected when participants were breathing evenly and steadily, including the resonant frequency (F_res_), impedance at 5 Hz (Z_5_), respiratory impedance at 5 Hz (R_5_). However, IOS tests was not included in two hospitals because IOS were not contained in the lung function equipment. Following the repeatability standards of lung function tests of the National Lung Health Education Program (NLHEP), results with a quality level lower than C were discarded.

Lung function parameters were as follows: forced expiratory volume in one second (FEV_1_), FEV_1_/FVC (forced vital capacity, FVC), FEV_1_%predicted (FEV_1_%pred), peak expiratory flow (PEF), forced expiratory flow at 50% (FEF_50%_), forced expiratory flow at 75% of exhaled forced vital capacity (FEF_75%_), maximal mid-expiratory flow (MMEF), diffusing capacity of the lungs for carbon monoxide (DLCO), and vital capacity (VC). The IOS parameters were: resonant frequency (F_res_), impedance at 5 Hz (Z_5_), respiratory impedance at 5 Hz (R_5_), respiratory impedance at 20 Hz (R_20_), and reactance at 5 Hz (X_5_).

### Air pollution and meteorological data collection

Daily records of PM_2.5_ in the southern and central districts were obtained from the Beijing Municipal Environmental Protection Bureau (http://www.bjepb.gov.cn/). Daily pollutant concentrations were obtained from the monitoring station nearest to each participant’s residential address. Daily estimates of PM_2.5_ data at the two monitoring stations were calculated as the 24-h mean concentration for the corresponding station. The mean distances from the residential address to the monitor station were 7.9 km (southern district) and 6.6 km (central district). Hourly meteorological data, including temperature and relative humidity were obtained from the China Meteorological Data Service Center (http://data.cma.cn/), including temperature and relative humidity (RH).

### Statistical analysis

Data were analyzed using R software (version 3.6.0) for Windows. Descriptive statistics were used to describe the general and clinical characteristics of the participants as mean ± standard deviation (SD) or number (percentage, %). The chi-square test and non-parametric Mann - Whitney U test (non-normal distribution) were used to examine the subgroup differences between the two regions. A generalized linear model (GLM) was used to estimate the associations between PM_2.5_, lung function and IOS parameters. We first performed single factor analyses to examine the associations between PM_2.5,_ lung function and IOS parameters. We then conducted further analyses to associate PM_2.5_ concentrations with lung function and IOS outcomes using a multiple linear regression model, adjusting for sex, smoking history, education level, age, body mass index (BMI), mean temperature, and relative humidity [19, 20]. The effect was evaluated on the day of the lung function tests (lag 0). The results are expressed as the value of lung function and IOS changes and, the 95% CI for increase in PM_2.5_. Statistical significance was set at *P* < 0.05.

This retrospective study followed the Strengthening the Reporting of Observational Studies in Epidemiology (STROBE) guidelines [21]. We tested for multicollinearity using the variance inflation coefficient (VIF) in the multiple regression model. In the present study, the VIF values were all close to 1 and less than 10. Multicollinearity was not observed [22].

## Result

### Summary for the participants characteristics and exposure parameters

During the 4-year study period, 1540 patients were included in the study. Of these, 185 cases were classified as having COPD comorbidity with asthma or other respiratory diseases, and seven patients had less information. A total of 1348 (87.53%) participants were selected for the final analysis, and divided into southern (694) and central (654) districts of Beijing. Most of the participants were men (70.77%). The proportion of patients with a history of smoking was 59.05%. The group with a low level education had significantly higher scores than the high-level education group (66.10%). The mean age was 66.88 ± 11.30 year. The mean body mass index (BMI) was 25.49 ± 3.96 kg/m^2^. The daily mean concentration of PM_2.5_ was 67.62 ± 18.21 µg/m^3^. The daily mean concentration of PM_2.5_ was with in the China Grade II standard for daily ambient air quality (75 µg/m³). Participants characteristics are shown in Table 1. There were no statistically significant differences between the two groups in terms of the clinical characteristics and exposure parameters (p > 0.05).

**Table 1:** Clinical characteristics and air pollution parameters of participants with COPD in Beijing, China

	southern district (N = 694)	central distinct (N = 654)	All (N = 1348)	^a*^ *P*
Clinical characteristics				
Sex, n(%)				
Male	472 (68.01)	482 (73.70)	954 (70.77)	0.386
Femal	222 (31.99)	172 (26.30)	394 (29.23)	
Smoking history, n(%)				
Never	278 (40.06)	274 (41.90)	552 (40.95)	0.125
Yes	416 (59.94)	378 (58.10)	794 (59.05)	
Education level, n(%)				
^b*^High level	216 (31.12)	241 (36.85)	457 (33.90)	0.063
^b^Low level	478 (68.88)	413 (63.15)	891 (66.10)	
^c^Age, year	63.37 (10.49)	70.61 (10.93)	66.88 (11.30)	0.112
^c^BMI, kg/m^2^	25.94 (3.65)	25.05 (4.18)	25.49 (3.96)	0.423
**Air pollution parameters**				
^c^PM_2.5,_ ug/m^3^	69.98 (19.06)	65.11 (16.92)	67.62 (18.21)	0.156
^c^Tem, ℃	10.03 (10.92)	12.07 (10.63)	11.02 (10.83)	0.174
^c^RH, %	51.08 (20.05)	50.60 (19.69)	50.85 (19.87)	0.268

**Abbreviations: BMI**, body mass index; **COPD**, chronic obstructive pulmonary disease; **PM**_**2.5**_, particulate matter with aerodynamic diameter ≤ 2.5 μm; **RH**, relative humidity; **Tem**, mean temperature

*<0.05; ^a*^Chi-square test and Non-parametric Mann-Whitney U test (non-normal distribution) were used to examine the subgroup differences

^b^Data on sex, smoking history and education level were presented as percentages (%)

^b*^High level: college degree or above; low level: senior high school and below

^c^Data of age, BMI, PM_2.5_, temperature and relative humidity were presented as mean ± SD (standard deviation)

The lung function and IOS parameters are presented in Tables 2 and 3, respectively. Compared to patients in the central district, those in the southern district had lower FEV_1_/FVC, FEV_1_%pred, PEF, FEF_75%_, MMEF and X_5_ (p < 0.05). Compared to patients in the central district, those in the southern district had higher F_res_, Z_5_ and R_5_ (p < 0.05).

**Table 2:** Pulmonary function parameters of participants in COPD patients in Beijing, China

	southern district (N = 694)	central distinct (N = 654)	All (N = 1348)	^a*^ *P*
FEV_1_/FVC, %	53.84 (10.74)	59.34(8.67)	56.67 (10.11)	< 0.001*
FEV_1,_ L	1.34 (0.58)	1.42 (0.60)	1.38 (0.59)	0.366
FEV_1_%pred, %	56.67 (19.77)	59.43 (20.75)	58.05(20.26)	< 0.001*
PEF, L/s	3.19 (1.52)	3.52 (1.55)	3.36 (1.54)	0.003*
FEF_50%_, %	23.55 (9.37)	24.04 (9.91)	23.80 (9.64)	0.193
FEF_75%_, %	22.75 (9.33)	24.07 (9.88)	23.41(9.61)	0.002*
MMEF, %	22.71 (10.22)	24.73 (9.31)	23.72 (9.77)	< 0.001*
VC, L	2.54 (0.86)	2.70(0.81)	2.62 (0.84)	0.673
DLCO, mmol/min/kpa/L	1.55 (2.59)	2.05 (1.30)	1.81 (1.95)	0.149

**Abbreviations: COPD**, chronic obstructive pulmonary disease; **DLCO**, diffusing capacity of the lungs for carbon monoxide ; **FEF**_**50%**_, forced expiratory flow at 50%; **FEF**_**75%**_, forced expiratory flow at 75% of exhaled forced vital capacity; **FEV**_**1**_, forced expiratory volume in one second; **FVC**, forced vital capacity; **MMEF**, maximal mid-expiratory flow; **PEF**, peak expiratory flow; **pred**, predict; **VC**, vital capacity

*P < 0.05; data of lung function parameters were presented as mean ± SD (standard deviation)

^a*^Non-parametric Mann-Whitney U test (non-normal distribution) were used to examine the subgroup differences

**Table 3:** IOS parameters of participants in COPD patients in Beijing, China

	southern district (N = 530)	central distinct (N = 347)	All (N = 877)	^a*^ *P*
F_res_, Hz	24.17 (6.33)	20.68 (7.29)	22.79 (6.94)	< 0.001*
Z_5_, kpa/(L/s)	0.88 (0.33)	0.66 (0.34)	0.78 (0.34)	0.025*
R_5_, kpa/(L/s)	0.61 (0.27)	0.57 (0.28)	0.59 (0.27)	0.008*
R_20_,kpa/(L/s)	0.44 (0.11)	0.35 (0.11)	0.35 (0.11)	0.147
X_5_, kpa/(L/s)	-0.34 (0.29)	-0.27 (0.23)	-0.31 (0.27)	0.038*

**Abbreviations: COPD**, chronic obstructive pulmonary disease; **F**_**res**_, resonant frequency; **IOS**, Impulse Oscillometry System; **R**_**5**_, respiratory impedance at 5 Hz; **R**_**20**_, respiratory impedance at 20 Hz; **X**_**5**_, reactance at 5 Hz; **Z**_**5**_, impedance at 5 Hz; *P < 0.05; data of IOS parameters were presented as mean ± SD (standard deviation) ;

^a*^Non-parametric Mann-Whitney U test (non-normal distribution) were used to examine the subgroup differences

### Effect on pulmonary function parameters and PM_2.5_

In the single factor analysis model, there were negative correlations between FEV_1_/FVC FEV_1_, FEV_1_%pred, PEF, FEF_75%_, MMEF and PM_2.5_ among patients with COPD.

**Fig. 2:**
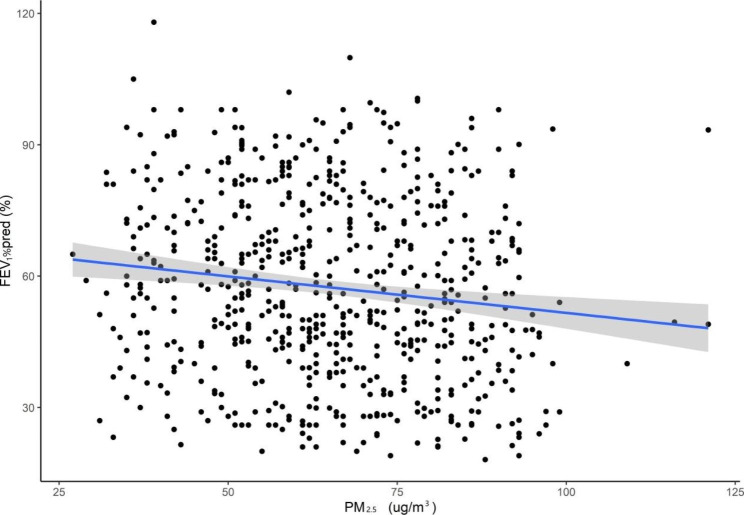
The exposure-response relationships between PM_**2.5**_ and FEV_1_%pred among patients with COPD in southern district in Beijing, China. **Abbreviations: COPD**, chronic obstructive pulmonary disease; **FEV**_**1**_, forced expiatory volume in one second; **PM**_**2.5**,_ particulate matter with aerodynamic diameter ≤ 2.5 μm; **pred**, predict 95% confidence bands (shaded areas)

**Fig. 3:**
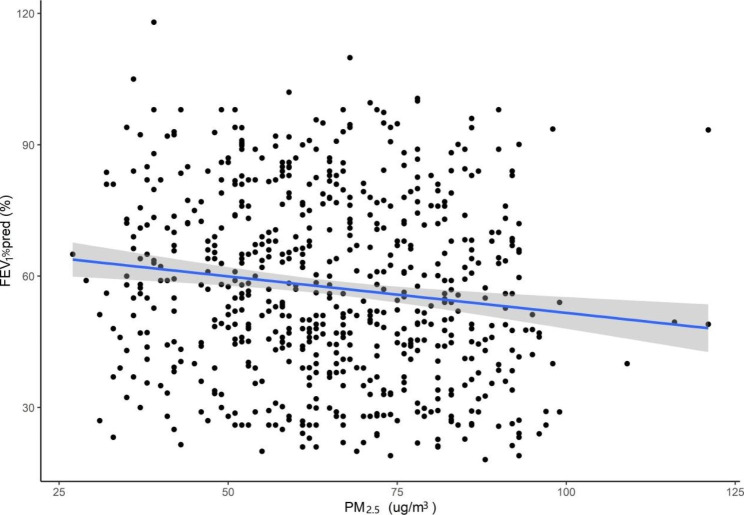
The exposure-response relationships between PM_2.5_ and FEV_1_%pred among patients with COPD in central district in Beijing, China. **Abbreviations: COPD**, chronic obstructive pulmonary disease; **FEV**_**1**_, forced expiatory volume in one second; **PM**_**2.5**,_ particulate matter with aerodynamic diameter ≤ 2.5 μm; **pred**, predict 95% confidence bands (shaded areas)

In the multiple linear regression model, we found a linear correlation between daily mean PM_2.5_ and lung function parameters (FEV_1_%pred, PEF and MMEF) (Table 4). An increased concentration of 10 µg/m^3^ in PM_2.5_ was associated with a decline of 2.448% (95% CI: −4.067 to − 0.831) in FEV_1_%pred, a decline of 204 ml/s (95% CI: −258 to − 49) in PEF, a decline of 0.973% (95% CI: −1.742 to − 0.203) in MMEF in the southern district, a decline of 1.886% (95% CI: −3.143 to − 0.638) in FEV_1_%pred, a decline of 188 ml/s (95% CI: −311 to -68) in PEF and a decline of 0.849% (95% CI: −1.665 to − 0.033) in MMEF in the central district. Figures 2 and 3 show the exposure-response relationships between PM_2.5_ and FEV_1_%pred in the southern and central districts of patients with COPD (taking FEV_1_%pred as an example).

**Table 4:** Effects of PM_2.5_ and lung function and IOS parameters among patients with COPD in Beijing, China (PM_2.5_--lag 0)

		β	SE	*P*	95% CI	VIF
**Lung function parameters, (N = 1348)**						
**Southern district**	FEV_1_%pred, %	−2.448	0.816	0.000*	−0.4.067,−0.831	1.672
	PEF, L/s	−0.204	0.047	0.005*	−0.258,−0.049	
	MMEF, %	−0.973	0.682	0.014*	−1.742, 0.203	
**Central district**	FEV_1_%pred, %	−1.886	0.642	0.003*	−3.143,−0.638	1.648
	PEF, L/s	−0.188	0.063	0.002*	−0.311,−0.068	
	MMEF, %	−0.849	0.422	0.043*	−1.665,−0.033	
**Total**	FEV_1_%pred, %	−2.083	0.050	0.000*	−3.047,−1.103	1.623
	PEF, L/s	−0.193	0.042	0.003*	−0.258,−0.043	
	MMEF, %	−0.932	0.301	0.002*	−1.518,−0.342	
**IOS parameters (N = 877)**						
**Southern district**	F_res_, Hz	0.814	0.402	0.006*	0.013, 1.589	1.641
	Z_5_, kpa/(L/s)	0.043	0.024	0.030*	0.002, 0.081	
	R_5_, kpa/(L/s)	0.051	0.023	0.029*	0.012, 0.073	
**Central district**	F_res_, Hz	0.548	0.243	0.028*	0.072, 1.033	1.749
	Z_5_, kpa/(L/s)	0.033	0.011	0.011*	0.014, 0.062	
	R_5_, kpa/(L/s)	0.028	0.010	0.004*	0.013, 0.049	
**Total**	F_res_, Hz	0.732	0.211	0.001*	0.313, 1.148	1.718
	Z_5_, kpa/(L/s)	0.036	0.012	0.013*	0.014, 0.057	
	R_5_, kpa/(L/s)	0.031	0.011	0.001*	0.002, 0.054	

**Abbreviations: COPD**, chronic obstructive pulmonary disease; **FEV**_**1**_, forced expiratory volume in one second; **F**_**res**_, resonant frequency; **IOS**, Impulse Oscillometry System; **MMEF**, maximal mid-expiratory flow; **PEF**, peak expiratory flow; **PM**_**2.5**_, particulate matter with aerodynamic diameter ≤ 2.5 μm; **pred**, predict; **R**_**5**_, respiratory impedance at 5 Hz; **Z**_**5**_, impedance at 5 Hz;

**β**, effect estimate; **SE**, standard error; **VIF**, variance inflation coefficient

*P-value < 0.05; **β** = regression coefficient of daily mean PM_2.5_ (10 µg/m^3^) concentration for lung function and IOS changes adjust for sex, smoking history,education level, age, body mass index (BMI), mean temperature, and relative humidity

Lag numbers indicate days prior to lung function tests (e.g. **lag0** is the day of performing)

**Fig. 4:**
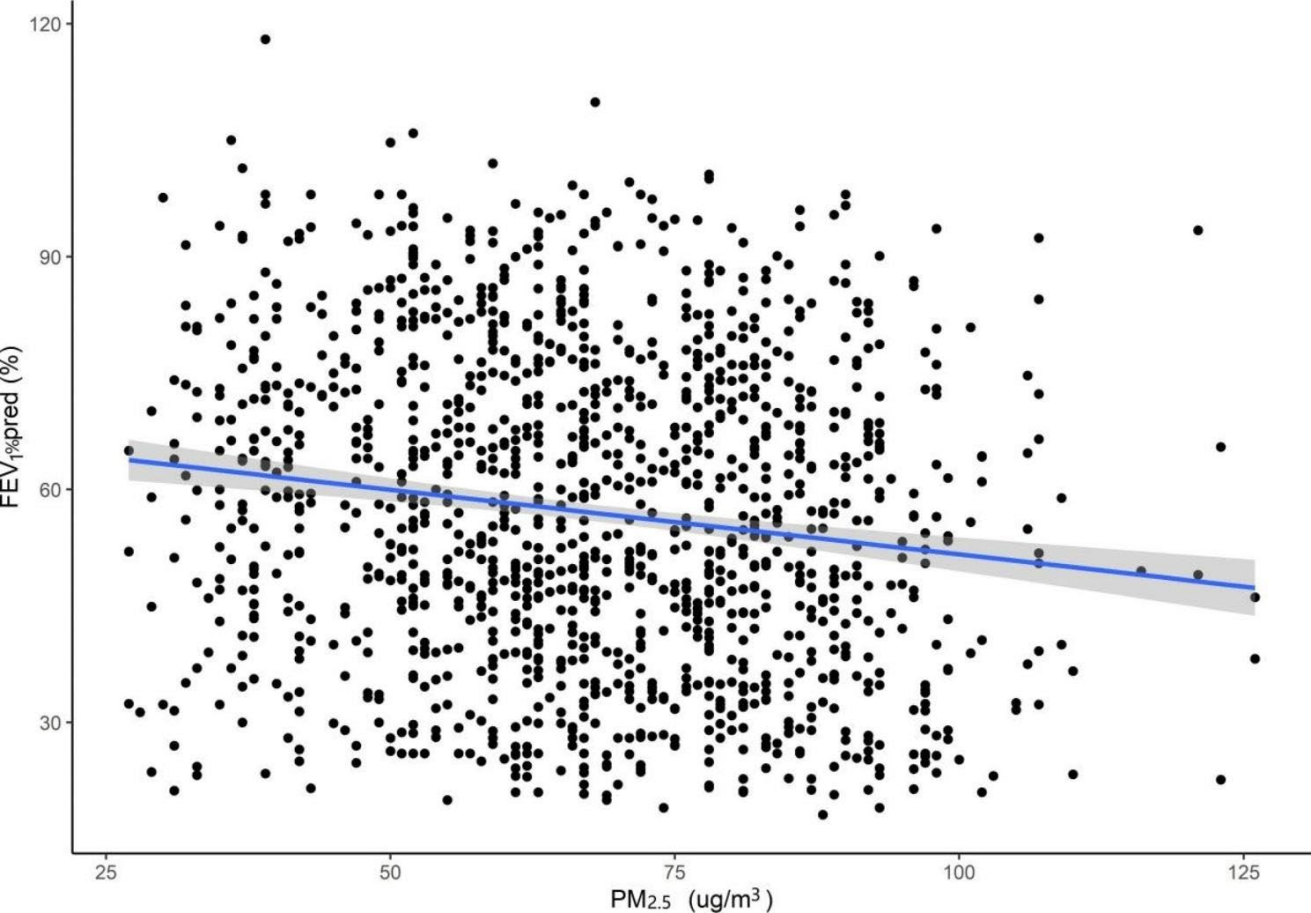
The exposure-response relationships between PM_**2.5**_ and FEV_1_%pred among patients with COPD in Beijing, China. **Abbreviations: COPD**, chronic obstructive pulmonary disease; **FEV**_**1**_, forced expiatory volume in one second; **PM**_**2.5**,_ particulate matter with aerodynamic diameter ≤ 2.5 μm; **pred**, predict 95% confidence bands (shaded areas)

In this study, we found that an increased concentration of 10 µg/m^3^ in PM_2.5_ was associated with a decline of 2.083% (95% CI: −3.047 to − 1.103) in FEV_1_%pred, a decline of 193 ml/s (95% CI: −0.26 to − 0.04) in PEF and a decline of 0.932% (95% CI: −1.518 to − 0.342) in MMEF in Beijing, China (Table 4). Figure 4 shows the exposure-response relationships between PM_2.5_ and FEV_1_%pred among patients with COPD in Beijing (taking FEV_1_%pred as an example).

### Effect on IOS parameters and PM_2.5_

In the single factor analysis model, there were negative correlations between X_5_ and PM_2.5_ and positive correlations between Fres, Z_5_, R_5_ and PM_2.5_ among patients with COPD.

**Fig. 5:**
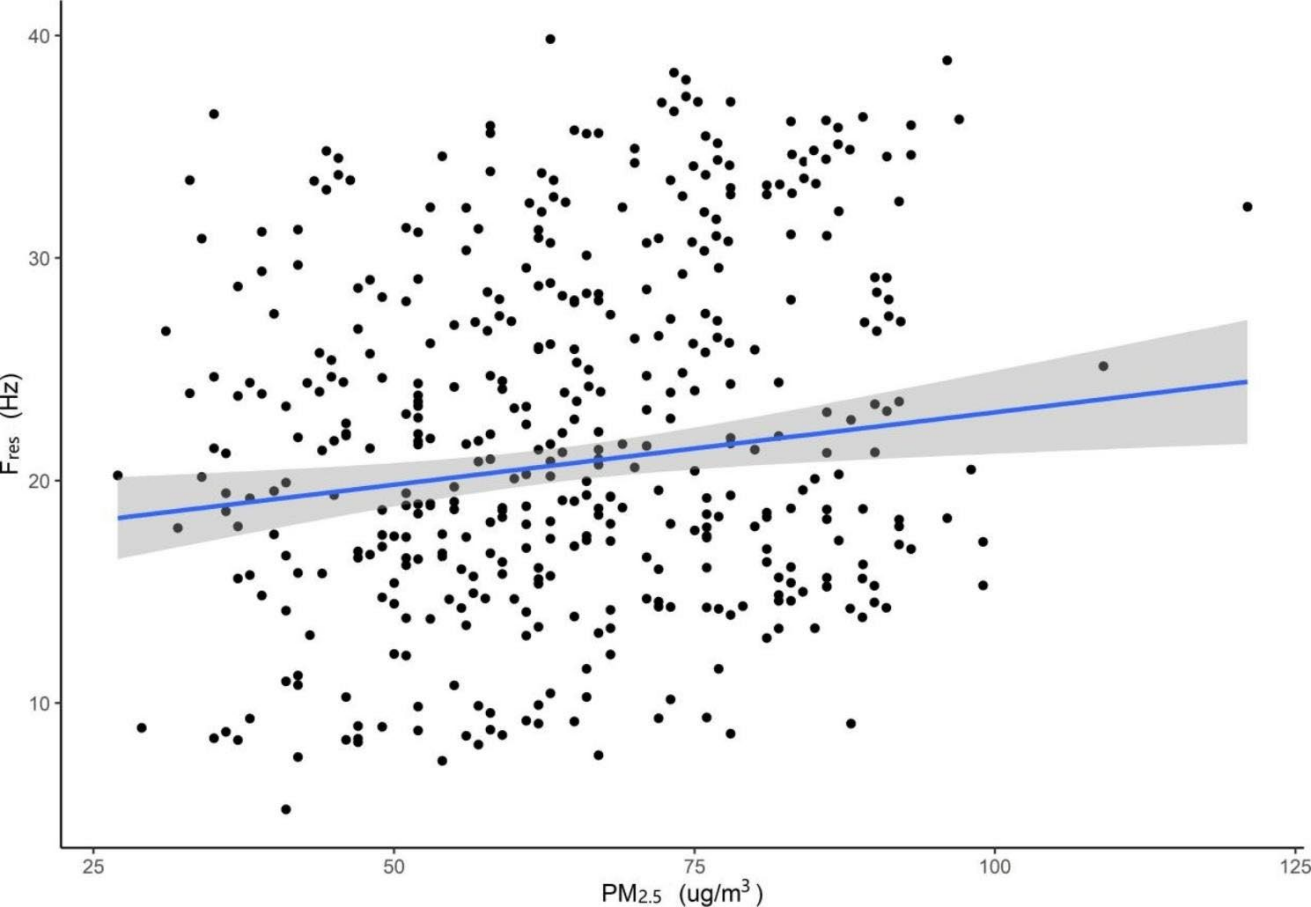
The exposure-response relationships between PM_**2.5**_ and F_res_ among patients with COPD in southern district, Beijing, China. **Abbreviations: COPD**, chronic obstructive pulmonary disease; **F**_**res**_, resonant frequency; **PM**_**2.5**,_ particulate matter with aerodynamic diameter ≤ 2.5 μm 95% confidence bands (shaded areas)

**Fig. 6:**
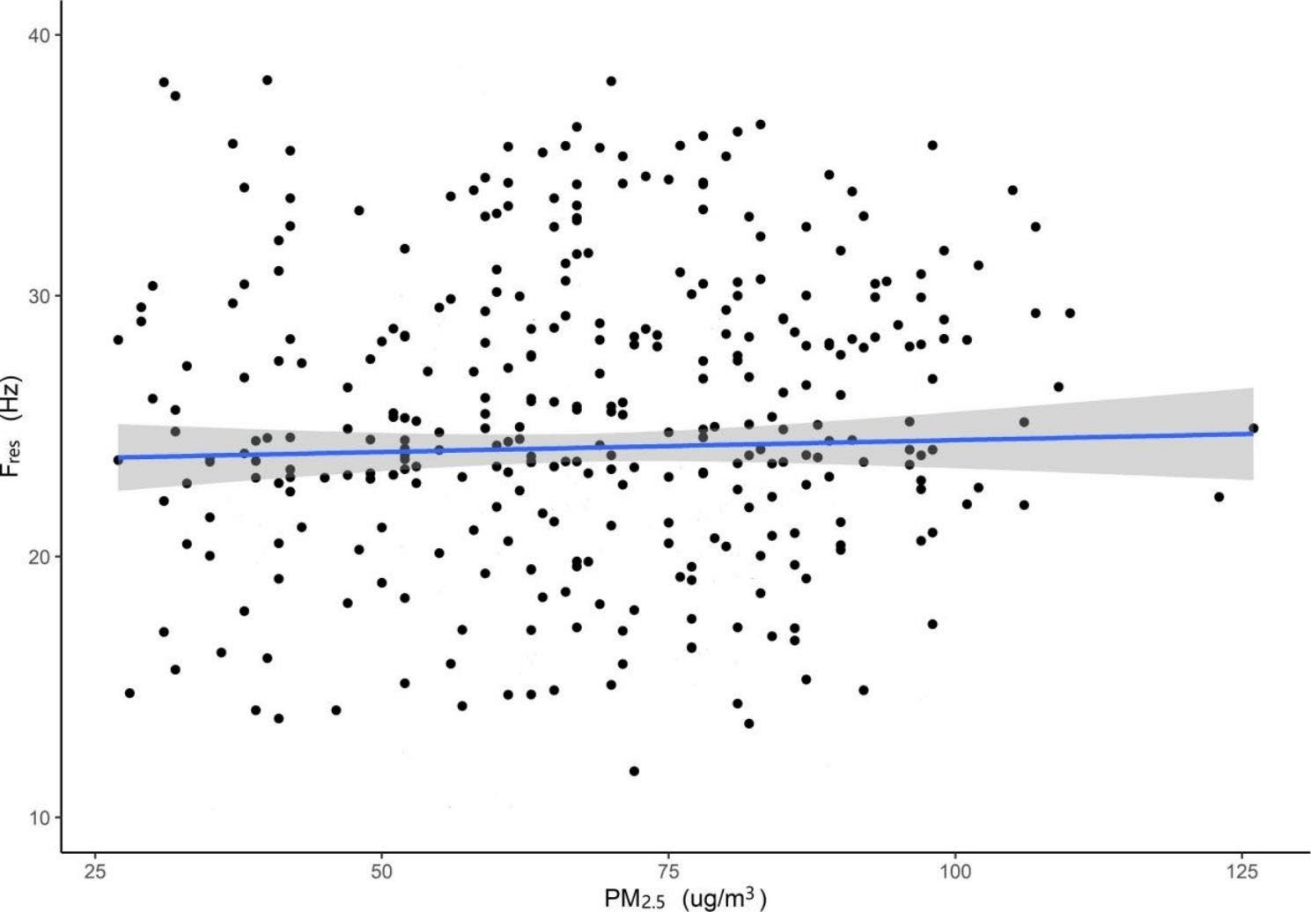
The exposure-response relationships between PM_**2.5**_ and F_res_ among patients with COPD in central district, in Beijing, China. **Abbreviations: COPD**, chronic obstructive pulmonary disease; **F**_**res**_, resonant frequency; **PM**_**2.5**,_ particulate matter with aerodynamic diameter ≤ 2.5 μm 95% confidence bands (shaded areas)

In the multiple linear regression model, we also found a linear correlation between daily mean PM_2.5_ and IOS parameters (F_res_, Z_5_ and R_5_) (Table 4). An increased concentration of 10 µg/m^3^ in PM_2.5_ was associated with an increase of 0.814 Hz (95% CI: 0.013 to 1.589) in F_res_, an increase of 43 kpa/(ml/s) (95% CI: 2 to 81) in Z_5,_ an increase of 51 kpa/(ml/s) (95% CI: 12 to 73) in R_5_ in the southern district, an increase of 0.548 Hz (95% CI: 0.072 to 1.033) in F_res_, an increase of 33 kpa/(ml/s) (95% CI: 14 to 62) in Z_5_ and an increase of 28 kpa/(ml/s) (95% CI: 13 to 49) in R_5_ in the central district. Figures 5 and 6 show the exposure-response relationships between PM_2.5_ and Fres in the southern and central district among patients with COPD (taking F_res_ as an example).

**Fig. 7:**
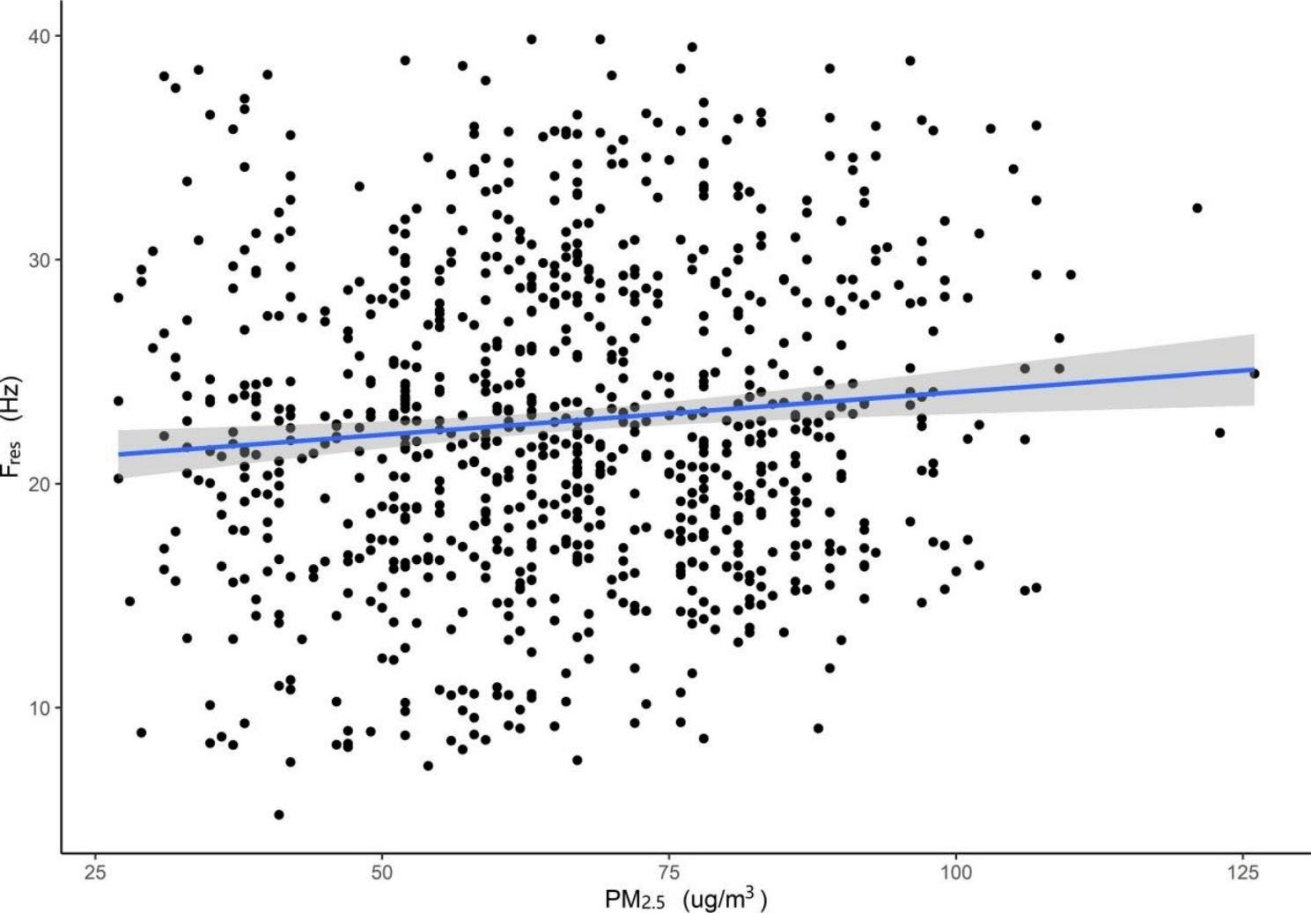
The exposure-response relationships between PM_2.5_ and F_res_ among patients with COPD in Beijing, China. **Abbreviations: COPD**, chronic obstructive pulmonary disease; **F**_**res**_, resonant frequency; **PM**_**2.5**,_ particulate matter with aerodynamic diameter ≤ 2.5 μm 95% confidence bands (shaded areas)

In this study, we found that an increased concentration of 10 µg/m^3^ in PM_2.5_ was associated with an increase of 0.732 Hz (95% CI: 0.313 to 1.148) in F_res_, an increase of 36 kpa/(ml/s) (95% CI: 14 to 57) in Z_5_, and an increase of 31 kpa/(ml/s) (95% CI: 2 to 54) in R_5_ in Beijing, China (Table 4). Figure 7 shows the exposure-response relationships between PM_2.5_ and Fres among patients with COPD in Beijing (taking F_res_ as an example).

**Fig. 8:**
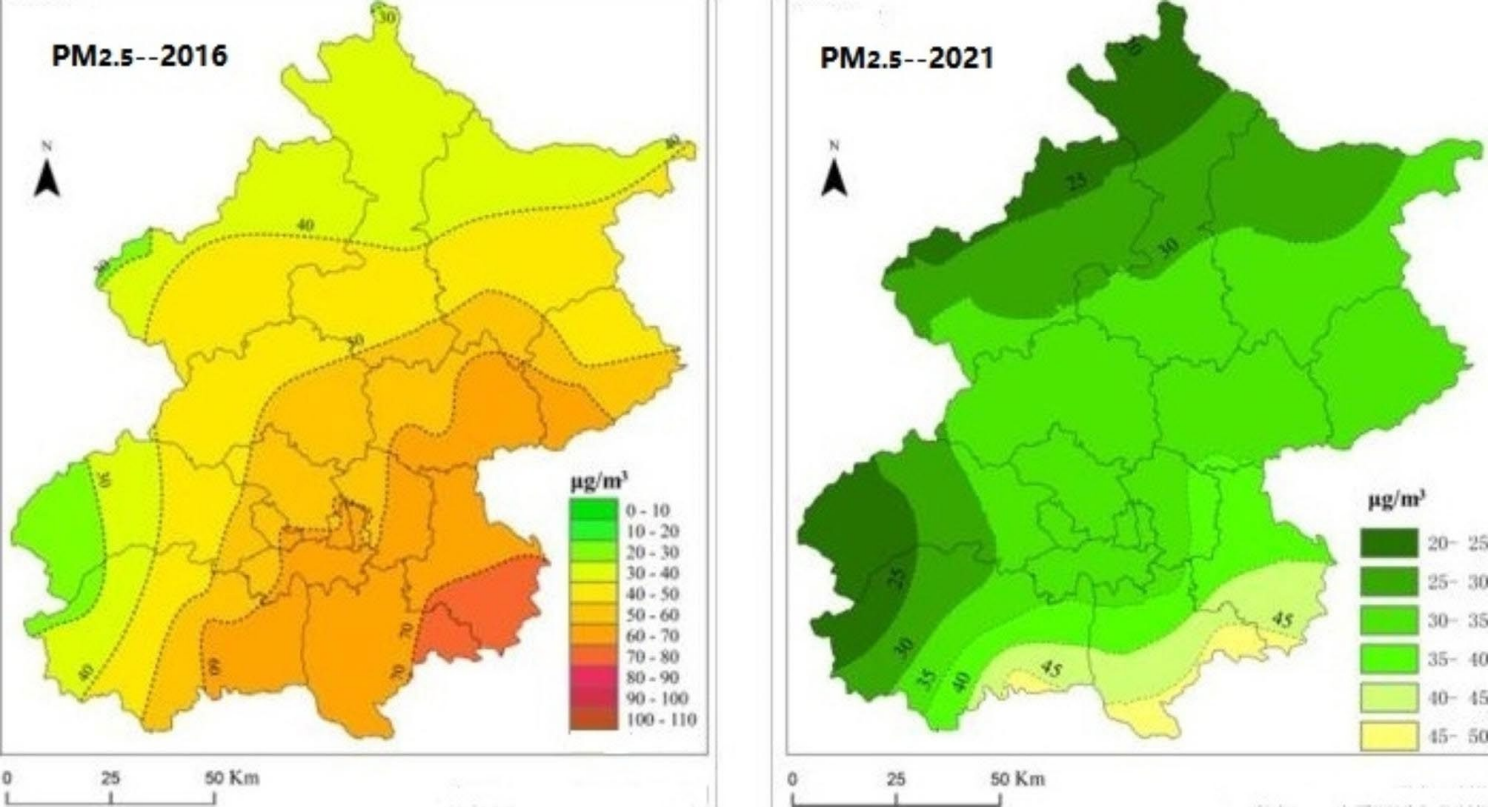
The changes of PM_**2.5**_ in Beijing, China (2016 and 2021). **Abbreviations: PM**_**2.5**_, particulate matter with aerodynamic diameter ≤ 2.5 μm

## Discussion

Exposure to ambient air pollution is associated with significant morbidity and mortality in COPD patients. In 2013 and 2020, our team conducted a meta-analysis on the association between PM_10_ / PM_2.5_ and COPD hospitalizations, and mortality. We found that a 10 µg/m^3^ increase in PM_10_ / PM_2.5_ was associated with a 2.7% (95% CI:1.9 to 3.6%) / 2.5% (95% CI:1.8 to 3.2%) increase in COPD hospitalizations and a 1.1% (95% CI: 0.8 to 1.4%) / 1.5% (95% CI:0.9 to 2.2%) increase in COPD mortality [23, 24]. These studies suggest that participation in air pollution can increase hospitalization and mortality in patients with COPD. Acute exacerbation or hospitalization for COPD tends to cause a sharp decline in lung function [25]. A decrease in lung function can lead to increased COPD mortality. Therefor the effects on particulate air pollution exposure and lung function should be taken seriously. However, the short term effects on ambient air pollution and lung function parameters remain unclear. The main purpose of this study was to assess the relationship among PM_2.5,_ lung function and IOS indices in patients with COPD.

To the best of our knowledge, this is the first large study to investigate the associations between short term exposure to ambient PM_2.5_ exposure with small airway dysfunction and IOS outcomes in adults with COPD. In this study, we found that compared to patients in the central district, those in the southern district presented with lower FEV_1_/FVC, FEV_1_%pred, PEF, FEF_75%_, MMEF and X_5_, and higher F_res_, Z_5_ and R_5_. This could be because the PM_2.5_ levels in the southern district of Beijing are heavier due to a massive and complex traffic flow and a large floating population. In this study, we confirmed the negative effects of short term exposure to PM_2.5_ on FEV_1_%pred but not on FEV_1_, FVC, or FEV_1_/FVC among patients with COPD. However, Duan et al. [12] found that in COPD patients, every 10 µg/m^3^ increase in PM_2.5_ exposure at lag2 was associated with a 14 ml (95% CI: −25 to − 3) decrease in FEV_1_ and a 25ml (95% CI: −50 to − 3) decrease in FVC. Ye et al. [26] demonstrated that short term exposure to PM_2.5_ was associated with a decrease in FEV_1_. For long term exposure to PM_2.5_, Liu et al. found that a 10 µg/m^3^ increase in PM_2.5_ concentrations was associated with a 0.96% (95% CI -1.65% to − 0.275) decrease in FEV_1_%pred [19]. Guo et al. found that every 10 µg/m^3^ increase in the 2-year average PM_2.5_ concentration was associated with a decrease of 2.94 ml (95% CI: −3:36 to − 2:51) in FEV_1_ in Hong Kong, China [27]. Dany et al. found that a 5 µg/m^3^ increase in PM_2.5_ concentration was associated with lower FEV_1_ (− 83.13 ml, 95%CI: −92.50 to − 73.75 ml) and FVC (− 62.62 ml, 95%CI: −73.91 to − 51.32 ml) [28]. The inconsistency in the findings of different studies may be attributable to differences in PM_2.5_, and population susceptibility. We also confirmed the negative effects of short term exposure of PM_2.5_ on PEF among patients with COPD. This findings is similar to the results of previous studies. Cortez-Lugo et al. found that a 10 µg/m^3^ daily average of a two-day personal exposure to PM_2.5_ was associated with a 0.02 L/s (95% CI−0.05 to−0.00) decrease in the morning and 0.05 L/s (95% CI−0.1 to−0.00) decrease at night in PEF in Mexico [29]. Yang et al. found that a 22.42 µg/m^3^ long-term exposure to PM_2.5_ was associated with a 0.08 L/s (95% CI−0.06 to−0.11) decrease in PEF in Beijing [30]. In this study, we found that the effect of short term exposure to PM_2.5_ and PEF was higher than those in previous studies. Aging and high severity of COPD may be attributed to the study results. The MMEF reflects the most effort-independent portion of the flow-volume curve and the most sensitive airflow to small airways, where chronic airflow obstruction is thought to originate [31]. We found that short-term PM_2.5_ exposure was significantly associated with MMEF, which is consistent with a previous study [26]. Guo et al. found that every 5 µg/m^3^ long-term exposure to PM_2.5_ was associated with a decrease of 1.65% in MMEF [32]. Bo et al. [33] found that every 5 µg/m^3^/year long-term exposure to PM_2.5_ was associated with a decrease of 70.22ml/s/year. We did not find clear associations between PM_2.5_, VC, and DLCO in either the single factor analysis model or the multiple linear regression model. Research on the association between PM_2.5_, VC and DLCO is limited. However, Duan et al. found that in COPD patients, every 10 µg/m^3^ increase in PM_2.5_ exposure at lag2, the DLCO decreased by−0.089 mmol/min/kPa (95% CI−0.156 to−0.023) [12]. Hou et al. found that an increase of 10 µg/m^3^ increase in the annual average PM_2.5_ exposure was associated with a decrease of VC by 89.12 ml (95% CI−124.94 to−53.3) [34]. VC and DLCO may be regarded as tools for the multidimensional assessment of COPD in the future.

Many previous studies have illustrated the harmful effects of PM_2.5_ on lung function in adults. However, the specific mechanism underlying PM_2.5_-induced hazards remains unclear. It is suggested that the exacerbation of inflammation, oxidative stress, immunosuppression, and the physical and chemical composition and origin of PM_2.5_ may play important roles in the decrease of lung function in COPD patients [35–37]. Guo et al.[38] indicated that short term exposure to PM_2.5_ was correlated with increased serum IL−2, IL−12, IL−17 A, interferon γ(IFN_γ_), and soluble CD40 ligand (sCD40L) among patients with COPD. This leads to inflammation and decreased FVC% prediction of COPD patients. Wang et al. [39] determined that the most toxic components of PM_2.5_ are metals, polycyclic aromatic hydrocarbons (PAHs), carbonaceous particles (CPs), and other organic compounds. PM_2.5_-induced cytokine release and oxidative stress are the main mechanisms leading to COPD. DNA damage and miRNAs can reduce lung function by promoting airway inflammation [40]. Our team will focus on the mechanism of PM_2.5_ exposure and lung function in the next step.

The Impulse Oscillometry System (IOS) can be used to assess small airway function, particularly in peripheral airway obstructive disease. COPD usually presents with small airway obstruction in the early stage, and it takes decades to develop slowly from small airway obstruction to FEV_1_ decline [41]. Fres is more sensitive in evaluate small airway obstruction than FEV_1_ in COPD patients [15, 42]. Cristiana et al. demonstrated that IOS can be used to detect early stage of COPD [43]. Frantz et al. reported that in asymptomatic and normal patients, IOS indicated higher pulmonary impedance and lower pulmonary reactance. This suggests that the IOS is more sensitive in detecting early changes in lung function [44]. In this study, there was no association between PM_2.5_ and FEV_1_ among patients with COPD, however, there was a significant association between PM_2.5_ and F_res_. Borrill et al. studied the IOS and specific airway conductance by comparing FEV_1_ in patients with COPD. The results showed that the IOS method performed better in identifying more delicate changes in pulmonary function than conventional lung function tests [45, 46]. This may be because there is a significant lag between the development of small airway disease and onset of FEV_1_ decline. FEV_1_ largely reflects atmospheric obstruction, and FEV_1_ may be abnormal when small airway lesions are present to a certain extent. In this study, we found positively correlations between PM_2.5_, Z_5_ and R_5_. The heavier the PM_2.5_ exposure was, the higher were the Z_5_ and R_5_. This reason may be because COPD patients have increased peripheral airway resistance caused by airway remodeling and scarring at an early stage. A previous study demonstrated that viscous resistance R can sensitively detect the early stages of COPD [47]. Xu et al. reported that long term variability of R_5_ was correlated with stable COPD [48]. In this study, there was no correlation between PM_2.5_ and X_5_. However, previous studies have demonstrated that X_5_ can be used to assess changes in lung compliance owing to airflow obstruction in patients with COPD. The more severe the COPD, the lower the X_5_ [49, 50]. Some studies have been conducted on the severity of IOS parameters and COPD [51, 52], while the effects on PM_2.5_ and IOS parameters are limited. Further detailed research is needed to address issue in the future.

Our study has several limitations. First, there may be other important, unknown, and unmeasured factors. For example, passive smoking, socioeconomic status, and time spent outside may have also played an important role in this study. Second, personal exposure to both indoor and outdoor air pollutants may not accurately represent general results. Finally, future health-related studies should use personal exposure data. This study has several strengths. First, we confirmed the actual residence and duration of residence of all patients by phone to eliminate the significant confounding effect of air pollution. Second compared to other studies, we analyzed more lung function indices. The last and the most, this study first focused on the correlation between PM_2.5_ and IOS index and demonstrated the effect of short-term PM_2.5_ exposure on COPD comprehensively.

The rapid development in Beijing has resulted in a significant increase in air pollution. The daily mean concentration of PM_2.5_ was with-in the China Grade II standard, was still higher than that reported in the USA and Europe. Fortunately, people have paid increasing attention to the effects of PM_2.5_ and we have observed changes in PM_2.5_ in Beijing through the efforts of the government (Fig. 8). In the future, more efforts are needed to deal with air pollution in China. Air pollution control will be a topic of priority in the future.

## Conclusion

In conclusion, this study suggests that short-term PM_2.5_ exposure may decrease lung function and increase the IOS indices in patients with COPD. The heavier the amount of PM_2.5_, the greater is the impact on lung function in each region. In addition, the results reported in this study suggest that Impulse Oscillometry System measurements in patients with COPD may provide a helpful tool for identifying adverse environmental impacts. Further detailed studies are needed to confirm these findings and to clarify the underlying mechanisms.

## Data Availability

All data generated or analyse during this study are included in this article. Further equipment can be directed to the corresponding author in email.
